# Multi-dendrite fragmentation dynamics in acoustic flow with oscillating cavitation bubbles revealed *in situ* by synchrotron X-ray radiography^☆^

**DOI:** 10.1016/j.ultsonch.2025.107684

**Published:** 2025-11-16

**Authors:** S. Wang, J. Kang, Z. Guo, K. Xiang, J. Wang, X. Li, M. Zou, J. Mi

**Affiliations:** a School of Materials Science and Engineering, Beijing Institute of Technology, Beijing, China; b School of Materials Science and Engineering, Tsinghua University, Beijing, China; c Key Laboratory for Advanced Materials Processing Technology, Ministry of Education, China; d Beijing Supreium Technology Co., LTD, Beijing, China; e School of Engineering and Technology, University of Hull, Cottingham Road, Hull HU6 7RX, UK; f Shanghai Synchrotron Radiation Facility, Shanghai Advanced Research Institute, Chinese Academy of Sciences, Shanghai 201204, China; g School of Materials Science and Engineering, Shanghai Jiao Tong University, Shanghai, China

**Keywords:** Dendrite fragmentation, Ga-In alloy, Ultrasound streaming, Ultrasonic cavitation bubbles, X-ray radiography

## Abstract

We used synchrotron X-ray radiography to study in-situ and in real-time the fragmentation dynamics of multiple dendritic microstructures in a Ga-40wt.% In alloy under ultrasound. A dedicated U-shaped quartz tube was designed and used as the sample holder to create well-controlled solidification conditions in a relatively large field of view suitable for imaging acoustic flow, cavitation bubbles, and their dynamic interactions with growing multiple dendrites. Without the presence of oscillating cavitation bubbles, the acoustic flow is able to bend multiple dendrites and then fragment them through cyclic fatigue mechanism over tens to hundreds of seconds. The local swirling flow, formed due to geometric constraints or flow obstacles, can trap and encircle multiple dendrites within the swirl, resulting in 3–4 times higher dendrite fragmentation efficiency than the linear streaming flow. The oscillating cavitation bubbles are more energetic and effective in causing mechanical fatigue fragmentation of dendrites, which often occur at the roots of dendrite arms in a time scale of ∼ 10 ms. Dendrite main trunks may also be fragmented by cavitation bubbles in a much longer time scale (i.e., a few seconds). The real-time observations have unambiguously revealed the fragmentation efficiency of multiple dendrites caused by different flow types and cavitation bubbles in the ultrasound processing conditions that are often found in industrial operations.

## 1 Introduction

Ultrasound melt processing (USMP) is an environmentally friendly and process-efficient physical field-based technique for microstructure refinement of metallic alloys in the solidification processes [[1], [2], [3], [4], [5], [6], [7]] without the need to add external grain refiners [1,5]. Ultrasonic cavitation (or cavitation-induced bubbles) and acoustic flow are the two most important and dominant dynamic phenomena often found in USMP. Up until the early 2010 s when the 3rd generation synchrotron X-ray sources had not become widely accessible tools for studying in situ and in real-time the metal solidification processes. Research activities concerning USMP of metallic alloys were typically carried out by numerical simulations or post-solidification microstructural characterization [[8], [9], [10]]. At first, instead of using liquid metal alloys directly, low-melting-point, organic transparent alloys, e.g., water-based camphor or succinonitrile alloys [[11], [12], [13], [14], [15]] were often used as the model alloys for imaging the dynamic interactions between cavitation bubbles, streaming flow, and the growing dendrites. For example, Shu *et al.* [11] were among the first to use optical high-speed imaging to capture the fragmentation of growing dendrites caused by the cavitation bubbles and shock waves produced at bubble implosion. In our previous research work using organic transparent alloys [17,18], we designed a special U-shaped quartz tube sample holder linked with two chambers. One is for accommodating the sonotrode tip and introducing ultrasonic waves. The other is for growing and then imaging the solidification microstructures. Such a design provided better control and an easily manageable, big view window for imaging the oscillating bubbles and streaming flow further away from the sonotorde tip. Using this setup, the dynamic phenomena of cavitation bubble interactions with dendrites, including cyclic fatigue bending, crack growth, and fatigue fragmentation of dendrites, were clearly revealed using 40,000 *fps* image acquisition speed [14]. The oscillation and implosion of cavitation bubbles were very effective in causing dendrite fragmentation [[15], [16], [17], [18]]. However, the physical properties of organic transparent alloys are very different from those of metallic alloys. Hence, in the past 20 years or so, high energy hard X-ray radiography and tomography techniques (lab based or synchrotron based) have been increasingly adopted in studying the solidification dynamics (melt flow and microstructural evolution) of metallic alloys in-situ and in real-time [[19], [20], [21]], especially for the solidification processes under external fields [[22], [23], [24], [25], [26]]. For example, Liotti *et al.* [24,25] studied pulse electromagnetic field solidification of an Al-15 %Cu alloy and the dynamics of Al dendrite fragmentation in different conditions. Nagira *et al.* [26] found that USMP can significantly enhance metal dendrite fragmentation. The solute transport in the mushy zones is greatly enhanced by the swirling flow and oscillation induced by ultrasonic pressure waves. However, those studies were conducted with a low imaging rate, i.e., a few to a few hundred frames per second, which cannot “see” the ms or µs time scale dynamic process of dendrite fragmentation. Recently, researchers in Mi’s group have used the ultrafast synchrotron X-ray imaging and tomography [[27], [28], [29], [30], [31], [32], [33], [34], [35], [36]] as well as the state-of-the-art X-ray Free Electron Laser MHz imaging [[36], [37], [38], [39]] technique to study the ultrasound cavitation dynamics and its interaction with structural and functional materials. In their work, imaging acquisition rates from hundreds of thousands to a couple of million frames per second have been routinely used to study the dynamics of bubble implosion and its effects on the microstructural evolution or layer exfoliation. However, due to the stochastic nature of the chaotic cavitation bubbles, it is still a great challenge (or with very low probability) to capture their interactions with growing dendrites in the same field of view under different ultrasonic conditions.

Here, we used the specially designed U-shaped quartz sample holder to study in real-time the dynamic interactions of the multiple dendrites of a Ga-40wt.% In alloy with acoustic flow and oscillating bubbles. Such an arrangement allows the dendrites to be grown in a precisely controlled manner in the left-hand side chamber, while the ultrasonic waves are introduced from the right-hand side chamber. In this way, the streaming flow developed further away from the sonotrode tip (i.e., the strong cavitation zone), and the associated oscillating bubbles can be transported to the large imaging view field in a much more controllable manner for visualization and imaging. Hence, significantly increasing the probability of capturing the dynamic interactions in-situ and in a large view field. The *in-situ* synchrotron X-ray radiography experiments were carried out at the X-ray Imaging and Biomedical Application Beamline (31124.02.SSRF.BLI3HB) of the Shanghai Synchrotron Radiation Facility (SSRF). The rich image datasets allow us to reveal and further elucidate the different fragmentation mechanisms and their relative importance in the ultrasound conditions that are more realistic and meaningful in industrial operations.

## 2 Experimental Methods

### 2.1 Alloys and experimental setup

**Fig. 1:**
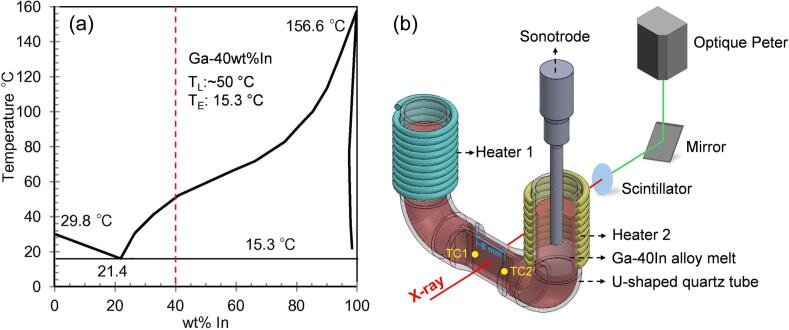
(a) The Ga-In binary alloy phase diagram, (b) the U-shaped quartz tube sample holder and experimental setup for X-ray imaging.

A low melting point alloy, Ga-40wt.% In, was used in the experiments. The alloy ingot was made by melting high-purity Ga (99.9 %) liquid and In (99.9 %) lumps with the correct weight ratio inside a graphite crucible in an electric resistance furnace under argon protection. The temperature was held at 150 °C for 30 min to homogenize the melt before casting into small ingots. The alloy liquidus is 50 °C, and the eutectic temperature is 15.3 °C according to the phase diagram in Fig. 1a [40]. The X-ray absorption contrast between the Ga and In phases is sufficient [[41], [42], [43], [44], [45], [46], [47]] for X-ray imaging.

Fig. 1b shows the specially designed U-shaped quartz tube used as the liquid alloy sample holder. It has a flat thin channel (∼0.3 mm thickness) in the horizontal middle section for X-ray imaging. The two vertical ends of the quartz tube have an inner diameter of 6 mm, big enough for inserting a sonotrode into the melt for applying ultrasound. The molten alloy was fed into the tube using a small diameter quartz tube connected to a syringe via a silicon rubber tube. Two K-type thermocouples (TC1 and TC2, both are 0.5 mm diameter) were placed at the two ends of the thin channel to monitor and record the temperature. Two small heaters were put at the two vertical ends of the quartz tube to melt the alloy and maintain a desired thermal gradient, which was realized by controlling the temperature of the hot end at 32.5 °C (heater 2), while the cold end was maintained at 25°C (heater 1). In such a condition, dendrite growth and the solidification front can be precisely controlled for obtaining high quality X-ray images.

The SONICS VCX 150 ultrasound device was used in the experiment. It has adjustable power settings from 0 W to 150 W with a fixed frequency of 20 kHz. The sonotrode diameter is 2 mm, immersed in the alloy melt ∼ 3 mm below the surface. The sonotrode was preheated with the alloy to the desired temperature before applying ultrasound [48,49]. The ultrasonic power used was 30 W and 45 W, respectively (20 % and 30 % of the full power setting). As the thin channel is further away from the sonotrode tip and the acoustic pressure is exponentially reduced, the streaming flow effect dominates at the thin channel section. Hence, a lower image acquisition rate with a larger view field can be used for imaging.

### 2.2 X-ray imaging parameters and image processing

The Ga-40wt.% In alloy was melted 3 times to homogenize the composition before any X-ray imaging experiments. A monochromatic X-ray beam of 37 keV was used, and the sample-to-scintillator distance was set at 20 cm. An Optique Peter camera was used with a 6.5 μm/pixel spatial resolution. The exposure time of each image was either 10 ms or 4 ms, i.e., an image acquisition rate of 100 *fps* or 250 *fps*. The field of view (FoV) was ∼ 3 × 2 mm.

All radiography images were processed using the open-source image processing software ImageJ (1.52a, NIH, USA) [50]. Firstly, all X-ray images were despeckled to reduce noise. Secondly, the despeckled images were averaged along the Z-axis projection to obtain the background image. Thirdly, the despeckled images were divided by the background image to obtain the normalized images. Finally, the brightness and contrast of the normalized images were auto-enhanced, and an unsharp mask filter (radius = 1.5 pixels, mask weight = 0.6) was applied to enhance the edge clarity.

**Fig. 2:**
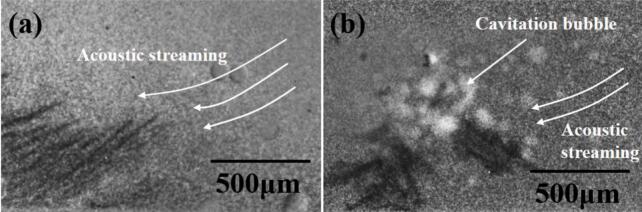
Two typical X-ray images, showing (a) the Ga-40wt.% In dendrites and the acoustic streaming flow (30 W case, see the corresponding Videos 1), (b) the Ga-40wt.% In dendrites and acoustic streaming flow as well as the cavitation bubbles (45 W case, see the corresponding Video 2).

Fig. 2a is a typical X-ray image showing the growing dendrites at the middle of the FoV (the 30 W case). Only acoustic flow was observed in this case. Fig. 2b shows the 45 W case, clearly showing the oscillating bubbles and streaming flow.

## 3 Results

### 3.1 Acoustic flow and swirling flow induced dendrite fragmentation

**Fig. 3:**
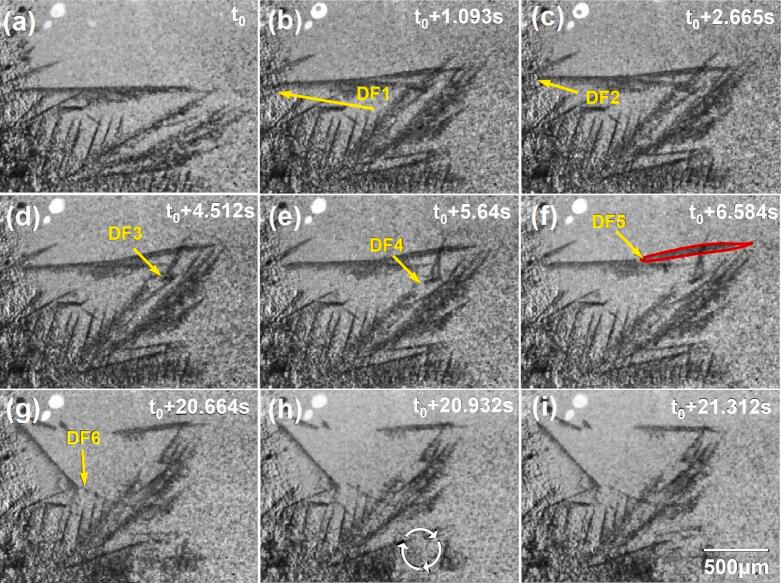
(a) − (f) dendrite arm fragmentation due to the cyclic fatigue effect induced by the acoustic flow. (g) − (i) dendritic fragmentation caused by the swirling flow. Experimental conditions: 30 W ultrasound, 1137.5 K/m thermal gradient, 250 fps (see Video 3).

Fig. 3 shows some typical multiple dendrite fragmentation events under the influence of acoustic flow. After the ultrasound (30 W) was switched on, a streaming flow was observed in the FoV, and some dendrites were seen to undergo cyclic bending under the streaming flow. The dendrites were also observed to collide frequently with adjacent ones. Many dendrite side arms (marked by the arrows in Fig. 3b-g) were fragmented by the fatigue effect imposed by the swirling flow. In 12 s of the applied ultrasound, a few sidearms (DF1-DF6) were fragmented. Fig. 3f also shows the remelting of some fragments due to the ultrasound induced heat effect as discussed later. A swirling flow appeared at the lower part of the FoV as indicated in Fig. 3h, churning clockwise in a circular region of ∼ 300 μm diameter, within which most dendrite arms were broken off in 0.02 s (see Fig. 3h-I and the vivid dynamic information in Video 3). The average velocity of the streaming flow was ∼ 5 mm/s. However, the linear velocity of the swirling flow in the melt reached ∼ 20 mm/s, calculated from the movement of the fragments in Video 3.

**Fig. 4:**
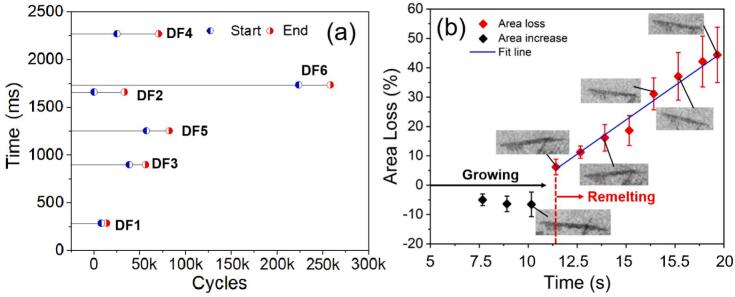
(a) The time needed to complete the dendrite fragmentation process, and (b) growing and remelting of the fragmented dendrite (marked in Fig. 3) under 30 W ultrasound.

From Video 3, the time to complete the dendrite fragmentation process (i.e., the moment when the fragmentation started until it ended) were extracted and shown in Fig. 4a. Actually, for most dendrites, the fragmentation process took thousands of ultrasound cycles (i.e., a few thousands of milli-seconds) to complete, much longer than that induced by bubble implosion [14]. Also, dendrite remelting (loss of dendrite area) was evident as indicated in Fig. 4b. The marked dendritic arm in Fig. 3f broke off due to the fatigue effect, vibrating and rotating clockwise with the acoustic flow. After fragmentation, the marked arms in Fig. 3f grew bigger as the thermal gradient was present in the melt.

However, after ∼ 1.8 s, the marked arms were remelted as the temperature increased due to the heat effect of the ultrasound. The length of the fragmented arm was gradually shortened from ∼ 700 μm to ∼ 450 μm, and part of its side arms disappeared as flushed continuously by the acoustic flow. The percentage of area loss versus ultrasound time is shown in Fig. 4b (the fragmented arm area in Fig. 3f was used as the starting reference). A few seconds after the ultrasound started, the dendrite area loss was negative (i.e., the dendrite was growing). However, dendrite remelting occurred as the acoustic flow continued to act on the dendrite. Fig. 4b shows that the area loss exhibited approximately a linear relationship versus time.

**Fig. 5:**
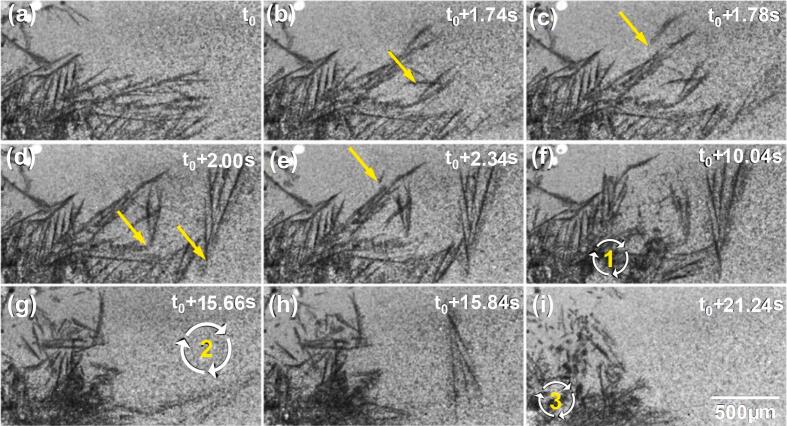
(a-e) Streaming flow induced dendrite fragmentation. (f-i) Swirling flow induced fragmentation. Experimental conditions: 30 W ultrasound, 1137.5 K/m thermal gradient, 250 fps (see Video 4).

Fig. 5 shows another case of multi-dendrite fragmentation. The acoustic flow fatigue effect bent over several dendrites after the ultrasound started. The arm marked by yellow arrow in Fig. 5b was fragmented first. Then many other arms were broken off (see Fig. 5c-e) under the continuous action of the swirling flow. Some of these arms were bent to a large angle before completely fracturing (see Fig. 5d). A local swirling flow region (marked 1 in Fig. 5f) was developed in the melt surrounded by the growing or fragmented dendrites. Many dendrite arms were fractured into small pieces due to melt stirring and phase collision. Some fragments flowed from the swirling flow region and into other melt regions. Interestingly, the swirling flow in region 1 damped out after the cavitation bubble disappeared (see Video 4). After ∼ 15.66 s of intensive stirring effect induced by the swirling flow, most of the large dendrites were broken into small fragments (see Fig. 5g) and moved to the left side of the view field, i.e., further away from the hot melt. Some fragments were carried away and moved out from the FoV. Then another swirling flow in region 2 appeared in the melt. However, no further fragmentation occurred because there were no visible dendrites near that swirling flow. As another cavitation bubble came into the FoV from the right, the third swirling flow in region 3 appeared (see Fig. 5h). Most of the nearby dendrites were broken into many more tiny fragments, as shown in Fig. 5i.

The X-ray images and videos also show that when a strong streaming flow changed direction because of a geometrical obstacle (sometimes because of the energetic oscillating bubbles), the streaming flow could turn into a strong swirling flow (see Video 4). Such swirling flow is much more effective in crushing more dendrites quickly, as illustrated in Fig. 5 and Video 4. This is because the linear velocity of the swirling flows (marked by 1–3 in Fig. 5) was ∼ 25 mm/s. In addition, the fragmentation phenomena also strongly depended on the location and time duration of the swirling flow.

### 3.2 Cavitation bubble induced dendrite fragmentation

**Fig. 6:**
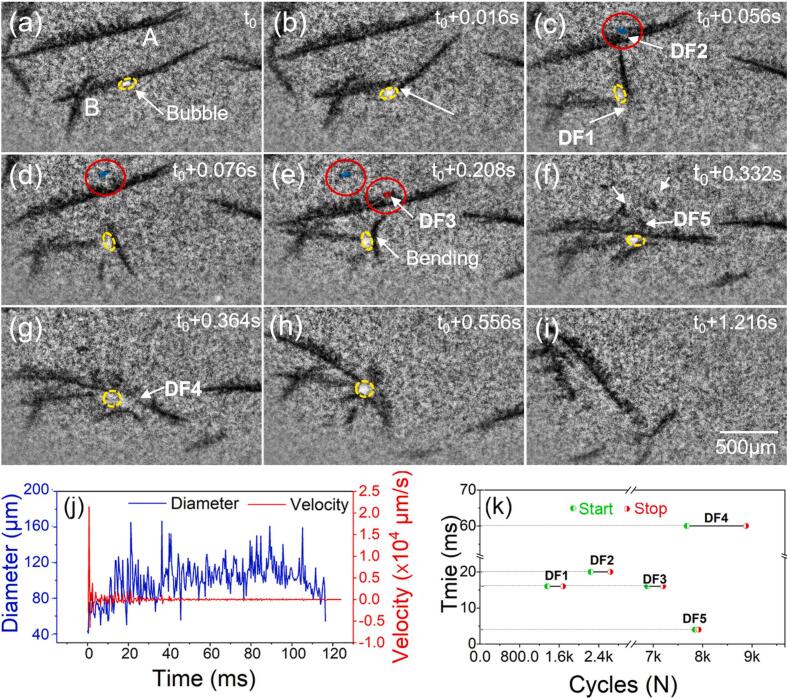
(a-i) A series of images that show clearly the oscillating bubbles induced dendrite fragmentation events. (j) The diameter and wall velocity of the marked bubble. (k) the time to complete the fragmentation of the marked dendrites. Experimental conditions: 30 W ultrasound, 1137.5 K/m thermal gradient, 250 fps (see Video 5).

**Fig. 7:**
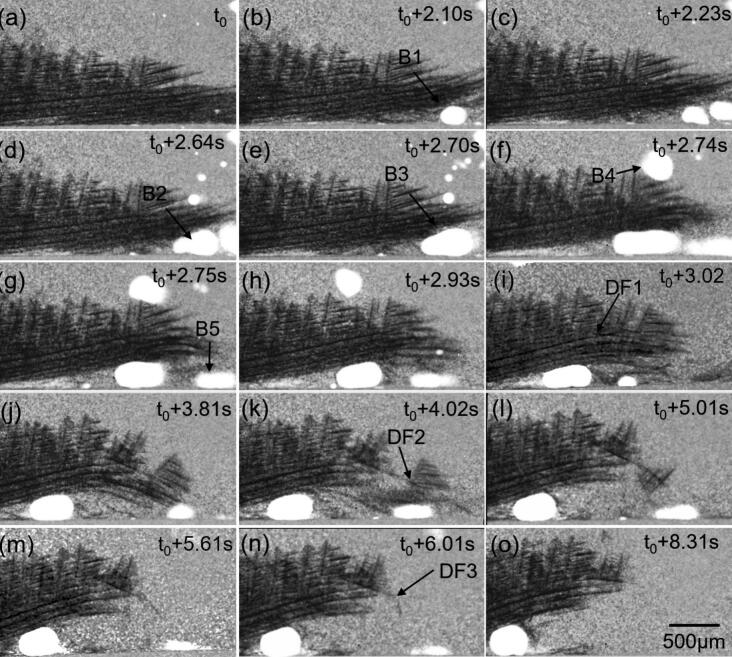
A series of X-ray images, showing multi-dendrites fragmentation induced by oscillating bubbles. Experimental conditions: 45 W ultrasound, 1137.5 K/m thermal gradient, 250 fps (see Video 6).

Fig. 6 (Video 5) shows the fragmentation of two dendrites (marked by A and B respectively) caused by the oscillating bubbles. A bubble of ∼ 40 μm diameter was vibrating onto the secondary arm of dendrite B (see Fig. 6a). The arm was fragmented after 0.01 s (see Fig. 6b) into two pieces (see Fig. 6, Fig. 6, Fig. 6). For dendrite A, its secondary dendrite arm was fractured at its root due to the vibration of a 20 μm diameter bubble (see Fig. 7, Fig. 7, Fig. 7). Dendrite A and B were seen to move closer (Fig. 6f), and then the trunk of dendrite A was fragmented into pieces (Fig. 6g-h). From Fig. 6h to Fig. 6i, the bubble imploded, shattered the dendrite fragments further, and dispersed them into the streaming flow.

Fig. 6j shows the bubble diameter evolution versus time and the corresponding velocity of the bubble wall. At the start, the abrupt increase in velocity resulted in immediate fragmentation of the dendrite trunk (see Fig. 6b). The bubble wall velocity damped out and reached a relatively constant value. Fig. 6k shows the time to complete the fragmentation events for all dendrites in approximately tens of ms. The small side arm took ∼ 20 ms (∼400 cycles) and the bigger primary trunk took ∼ 60 ms (∼1200 cycles).

The initial bubble near dendrite B was left over from the previous experiment with 45 W ultrasound power. All dendrites' arms fractured at the root. Still, the dendrite trunk was also found fragmented at the region where the oscillating bubble touched, indicating the effectiveness of the oscillating bubbles on dendrite fragmentation. In addition, the time of oscillating bubbles induced fragmentation was shorter than that caused by streaming flow.

### 3.3 The effectiveness of oscillating bubbles induced multi-dendrites fragmentation

Fig. 7 and Video 6 show oscillating bubbles induced multi-dendrites fragmentation. Fig. 7a shows the solidified In dendrites (dark phase) in the lower half of the FoV, which grew from left to right (∼2 mm long). The gray area above was the melt. Bubble B1 (250 μm diameter) moved into the FoV (see Fig. 7b), “squeezing” into the gap between the quartz tube and the solidified dendrites, driven by the ultrasonic stream, causing the dendrites to bend upwards. Then more bubbles moved in and coalesced with bubble B1 to form a bigger bubble B2 and finally bubble B3 (∼500 μm diameter, see Fig. 7d-e). At the same time, many other smaller bubbles (with a size of 10–100 μm in diameter) were also transported into the FoV by the streaming flow. They vibrated strenuously and gradually merged into a big bubble B4 (see Fig. 7e-f) and then bubble B5. They vibrated energetically at the dendrite tip.

Due to geometry constraints, bubble B3 was restrained and only vibrated slightly, and therefore did not cause any obvious fragmentation. On the contrary, bubble B4 was free in the melt zone, moved away without touching any dendrites (see Fig. 7g-i). Bubble B5, however, was near the dendrite tip in the free melt and vibrated violently. There was sufficient acoustic energy for Bubble B5 to go through the split-and-coalescence process, which further fragmented many small dendrites (see Fig. 7g-n). For example, dendrite fragmentations (DF1-DF3) occurred as indicated by the black arrows in Fig. 7, Fig. 7, Fig. 7. The sufficient energy allows bubble B5 to “punch” through all solidifying dendrites and finally move away with the stream flow.

## 4 Discussions

### 4.1 The effect of swirling flow on dendrite fragmentation

Many previous studies have investigated the acoustic flow's effect on dendrite fragmentation [29,[51], [52], [53]]. The main mechanisms proposed are ultrasonic flow-induced thermal fluctuation and mechanical fragmentation [29]. Dendrite arm remelting occurs due to the thermal fluctuation driven by streaming flows. The fragments then act as the seeds of new grains, leading to grain refinement. This study shows that strong swirling flow adequately fragments the dendrites within its effective area (Fig. 3 and Fig. 5).

**Fig. 8:**
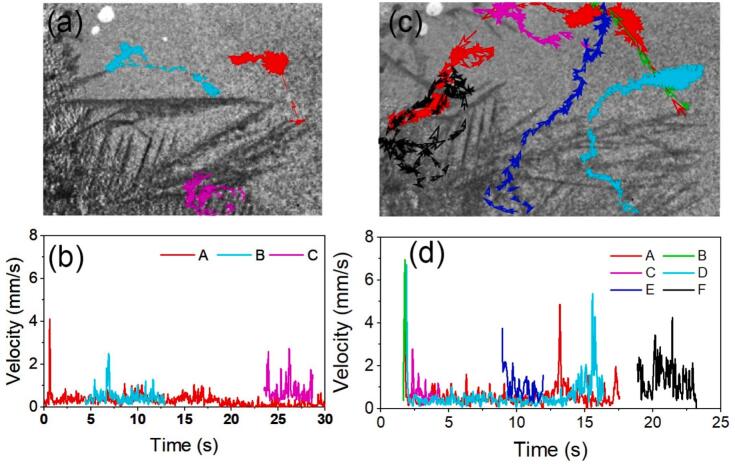
(a-b) shows the moving tracks and velocities of the selected dendrites in Fig. 3 (also see Video 7). (c-d) shows the moving tracks and velocities of the selected dendrites in Fig. 5 (see Video 8).

The velocities of the moving dendrites and tracks in Fig. 3 and Fig. 5 were extracted using Tracker [54], and the results are shown in Fig. 8. The moving tracks of A, B, and C were tracked every 10 frames, and all the trace routes are rendered with arrows in different colour as shown in Fig. 8a. The moving tracks of dendrites A and B are approximately linear. However, dendrite C shows a circular pattern, indicating the formation of swirling flow in this area. The velocities of dendrites A and B varied slightly (in a range of 0–1 mm/s) except at the very start (4 mm/s and 2 mm/s respectively, see Fig. 8b). However, the velocity of dendrite C varied in 1–3 mm/s in most of the moving trace, indicating that the swirling flow has more energy that can impact onto the dendrites, leading to higher efficiency in fragmentation.

The moving tracks of the moving dendrites in Fig. 5, marked as A-F, and their velocities, were extracted (see Fig. 8, Fig. 8). The moving tracks of dendrites B and C are similar, with both smooth traces under ultrasonic flow with velocity varied slightly (in a range of 0–1 mm/s) except at the very start. There is swirling flow in the moving tracks of dendrites A, D, E, and F (see in Fig. 8c). The velocities of the mentioned dendrites above are higher during the swirling flow phase (see Fig. 8d). Dendrites A, D, and E are similar in that they all go through a single period of swirling flow. Still, they do so at different stages (A middle, D end, E start). At a comparatively steady state, the velocities of dendrites A, D, and E varied slightly (between 0 and 1 mm/s). But in the swirling flow, the velocities rose to 4 mm/s to 5 mm/s (see Fig. 8d). Dendrite F experienced more energy impact because it was in the swirling flow (see Fig. 8d), which resulted in more effective fragmentation because the stresses from the swirling flow were greater (see the supplementary material) than those from the streaming flow.

### 4.2 The effect of cavitation bubbles on dendrite fragmentation

Ultrasonic cavitation bubbles play an essential role in dendrite fragmentation. The shock waves generated at bubble implosion were considered the most effective in dendrite fragmentation [14,27]. Also, the cyclic fatigue force due to the oscillating bubbles [17] is another important factor [15]. In our research, the dendrites were present in the area far from the sonotrode tip (i.e., the cavitation zone); hence, the observed bubbles were seen to oscillate quasi-statically in relatively long time without implosion. This explains why the dendrites in Fig. 6 and Fig. 7 were mainly caused by the fatigue effect of oscillating bubbles in a few thousand ultrasound cycles.

**Fig. 9:**
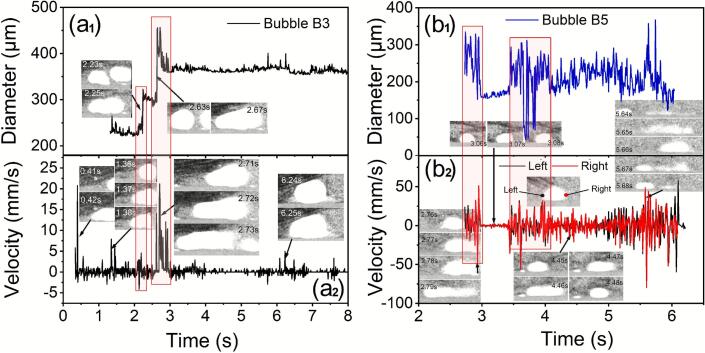
The equal diameter and velocity of bubbles 3 and 5 in Fig. 7. Fig. 9a shows the coalescence process of small bubbles (a1) and the corresponding velocity, Fig. 9b shows the violent vibration of bubble 5 (b1) together with their velocities of bubble walls (b2).

**Fig. 10:**
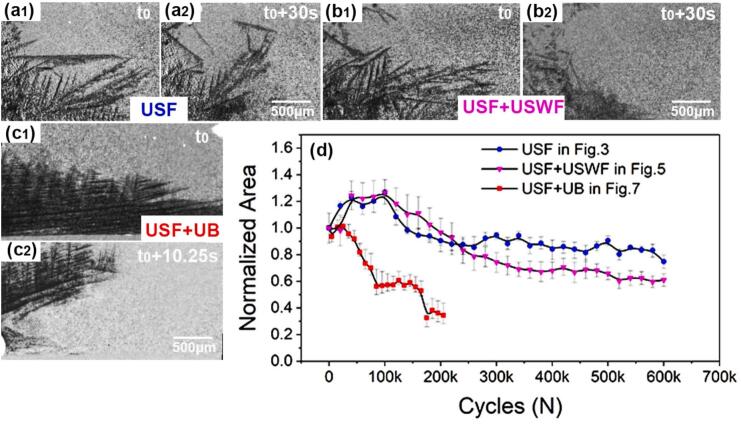
The normalized area changes due to dendrite fragmentation. (a1-a2) two typical images selected from Fig. 3, and the area change under ultrasonic streaming flow (USF) is indicated by the blue dots in (d). (b1-b2) Two typical images from Fig. 5, and the area change under ultrasonic streaming flow (USF) plus ultrasonic swirling flow (USWF) is indicated by the magenta triangles in (d). (c1-c2) Two typical images selected from Fig. 7, and the area change under streaming flow plus cavitation bubbles (USF + UB) are indicated by the red squares in (d). (d) The normalized area evolution for the three cases.

The vibration amplitude of the bubbles in Fig. 7 was measured, and Fig. 9 shows the corresponding equivalent diameter and the bubble wall velocities. Fig. 9a_1_ shows the diameter evolution of bubble B3 (amplitude of ∼ 10 μm at steady state) and the corresponding velocity of the bubble wall (Fig. 10a_2_) in Fig. 7. The bubble wall velocity was in the range of 5-20 mm/s except at the specific point. This is partly due to the bubble entering a geometrically constrained region in the FoV, and partly because the transmission of the acoustic pressure was somehow “blocked” by the dendrites or other geometrical barrier. Such vibration only has a minimal effect on the dendrite fragmentation (see Fig. 7). In addition, bubbles' coalescence can cause a sudden change in bubble wall velocities, as evidenced in bubble B3 and B5, as shown in the marked data in Fig. 9.

Bubble B5 has a much higher vibration amplitude (see Fig. 9b), wiht an equal diameter of 150 ∼ 300 μm (Fig. 9b_1_). The bubble velocity was at −50 ∼ 50 mm/s as shown in Fig. 9b_2_. Most of the dendrites’ fronts were fragmented by the violent vibration of bubble B5. It should be noticed that bubble B5 (at 3 ∼ 3.5 s in Fig. 10b) has a small diameter (∼150 μm) and a minimum velocity (∼0), indicating that the vibration of bubble B5 was restricted under some fragmented dendrites (see Fig. 7l-m). At such a lower amplitude level, almost no extra dendrites were fragmented. The instant velocities of bubbles B3 and B5 can reach as high as 20 mm/s and 50 mm/s, respectively. These values are much higher than those of acoustic flow and swirling flow. From the information shown in Fig. 7 and Fig. 10, it is very clear that the bubble’s vibration amplitude is a dominant parameter in causing dendrite fragmentation.

### 4.3 The dominant mechanisms in causing dendrite fragmentation

Dendrite fragmentation due to ultrasound has been widely accepted; cavitation bubble implosion, oscillation, and acoustic streaming flow can all cause dendrite fragmentation. This study concentrated on the region far from the cavitation zone (the cavitation bubble cloud zone). We calculated the fragmentation events that occurred in Fig. 3 (Video 3), Fig. 5 (Video 4) and Fig. 7 (Video 6), and the statistical results are shown in Fig. 10d. (please note that, in Fig. 10, a1 and a2 are two typical images selected from Fig. 3; b1 and b2 are those from Fig. 5; c1 and c2 are from Fig. 7). Clearly, the area loss (i.e., the fragmentation efficiency) under the cavitation bubbles is much higher than that caused by the streaming or swirling flow. More importantly, the streaming flow effects were weak in terms of causing dendrite fragmentation but lasted a substantially longer than that of the cavitation bubbles (approximately 6 times longer in the cases here). However, there is a linked and coupled phenomenon in that the streaming or swirling flow is the “carrier” to transport the cavitation bubbles to the dendrite regions.

## 5 Conclusions

We studied *in-situ* multiple dendrite fragmentation dynamics of a Ga-40wt.% In alloy under different ultrasound conditions by synchrotron X-ray radiography. We used a unique experimental apparatus to visualize multiple dendrite fragmentation dynamics induced by acoustic flow and cavitation bubbles. The fragmentation phenomena are different from those occurred in the intensive cavitation bubble cloud zone. The important findings of this research are:

1. The acoustic flow and swirling flow can create cyclic fatigue actions on the dendrites, causing the dendrites to be fragmentated by fatigue over a relatively longer period of time. Such fragmentation may also be enhanced by the remelting effect of the hot melt carried by the ultrasonic flow, especially when a strong swirling flow is formed due to any geometrical constraint in the path of the streaming flow.
2. The energetic and vibrating cavitation bubbles are more effective and efficient in causing mechanical fatigue fragmentation of dendrites, which often occurs at the dendrites' arm roots. The cavitation bubbles may also fragment the dendrite trunk, but occur over much longer ultrasonic cycles.

## CRediT authorship contribution statement

**S. Wang:** Writing – review & editing, Writing – original draft. **J. Kang:** Writing – review & editing. **Z. Guo:** Investigation. **K. Xiang:** Formal analysis, Data curation. **J. Wang:** Writing – review & editing. **X. Li:** Writing – review & editing. **M. Zou:** Writing – review & editing. **J. Mi:** Writing – review & editing, Funding acquisition.

## Declaration of competing interest

The authors declare that they have no known competing financial interests or personal relationships that could have appeared to influence the work reported in this paper.
