# Supplementary material for: Multi-dendrite fragmentation dynamics in acoustic flow with oscillating cavitation bubbles revealed in situ by synchrotron X-ray radiography
Source: Ultrason Sonochem. 2025 Nov 16;123:107684. doi: 10.1016/j.ultsonch.2025.107684 (PMC12686920; doi:10.1016/j.ultsonch.2025.107684)
Supplement: Supplementary Data 1 [file mmc1.docx]

**Multi-dendrite fragmentation dynamics in acoustic flow with oscillating cavitation bubbles revealed in situ by synchrotron X-ray radiography**

**Supplementary Materials**

S. Wang^1^, J. Kang^2, 3^, Z. Guo^4^, K. Xiang^5^, J. Wang^6^, X. Li^1^, M. Zou^1*^, J. Mi^5, 7*^

^1^ School of Materials Science and Engineering, Beijing Institute of Technology, Beijing, China

^2^ School of Materials Science and Engineering, Tsinghua University, Beijing, China

^3^ Key Laboratory for Advanced Materials Processing Technology, Ministry of Education, China

^4^ Beijing Supreium Technology Co., LTD, Beijing, China

^5^ School of Engineering and Technology, University of Hull, Cottingham Road, Hull, HU6 7RX, UK

^6^ Shanghai Synchrotron Radiation Facility, Shanghai Advanced Research Institute, Chinese Academy of Sciences, Shanghai 201204, China

^7^ School of Materials Science and Engineering, Shanghai Jiao Tong University, Shanghai, China

^*^ Corresponding author: [zoums@bit.edu.cn](mailto:zoums@bit.edu.cn) (M. Zou), J.Mi@hull.ac.uk (J. Mi)

To determine the stresses of swirling flow on dendrites arms, a model developed by Pilling and Hellawell [1] was used. The stress for a flow with velocity *v* passing across a cylindrical dendrite with length *L* and radius *r* can be calculated by equation (S1).

$\sigma=\frac{6\mu vL^{2}}{r^{3}}$ (S1)

Where σ is the stress acting on the arm, *µ* is the dynamic viscosity, *r* is the radius of dendrite root, *L* is the dendrite length. The value of stress is directly proportional to the velocity under similar dendrite, The velocity for pure ultrasonic streaming flow is ~1-5 mm/s, the linear velocity of swirling flow can reach 5~25mm/s. Thus, the stresses caused by the swirling flow is about 5 times higher than that by the streaming flow.

**References:**

1. Kotadia H R, Qian M, Eskin D G, et al. On the microstructural refinement in commercial purity Al and Al-10 wt% Cu alloy under ultrasonication during solidification[J]. Materials & Design, 2017, 132: 266-274.
